# The Implication of Oxidative Stress and AMPK-Nrf2 Antioxidative Signaling in Pneumonia Pathogenesis

**DOI:** 10.3389/fendo.2020.00400

**Published:** 2020-06-17

**Authors:** Weitong Xu, Tingting Zhao, Hengyi Xiao

**Affiliations:** Lab for Aging Research, State Key Laboratory of Biotherapy, National Clinical Research Center for Geriatrics, West China Hospital, Sichuan University, Chengdu, China

**Keywords:** AMPK, Nrf2, pneumonia, oxidative stress, diabetes, obesity

## Abstract

It is widely recognized that chemical, physical, and biological factors can singly or synergistically evoke the excessive production of oxidative stress in pulmonary tissue that followed by pulmonary lesions and pneumonia. In addition, metabolic and endocrine disorder-induced diseases such as diabetes and obesity often expressed higher susceptibility to pulmonary infections, and presented severe symptoms which increasing the mortality rate. Therefore, the connection between the lesion of the lungs and the metabolic/endocrine disorders is an interesting and essential issue to be addressed. Studies have noticed a similar pathological feature in both infectious pneumonia and metabolic disease-intercurrent pulmonary lesions, that is, from the view of molecular pathology, the accumulation of excessive reactive oxygen species (ROS) in pulmonary tissue accompanying with activated pro-inflammatory signals. Meanwhile, Adenosine 5′-monophosphate (AMP)-activated protein kinase (AMPK) and nuclear factor erythroid-2-related factor 2 (Nrf2) signaling plays important role in metabolic/endocrine homeostasis and infection response, and it's closely associated with the anti-oxidative capacity of the body. For this reason, this review will start from the summary upon the implication of ROS accumulation, and to discuss how AMPK-Nrf2 signaling contributes to maintaining the metabolic/endocrine homeostasis and attenuates the susceptibility of pulmonary infections.

## Introduction

Pneumonia is one of the most common clinical problems in human beings, characterized by the inflammation in the terminal airway, alveoli, and lung mesenchyme, usually caused by pathogen infection such as bacteria, viruses, fungi, and mycoplasma (1). If uncontrolled, pulmonary inflammation will progress to serious respiratory dysfunction and mortality especially in child and elderly people (2–4). Although amounts of antibiotics have been applied, the mortality rate of pneumonia has increased due to the rising drug resistance.

The outcome of pneumonia is closely associated with the individual's basic diseases. For example, patients with chronic obstructive pulmonary disease who smoke or inhale irritant gas for a long time have a high risk of pneumonia due to the irreversible decline in pulmonary function (5–8). Clinical data also have presented that pneumonia is a threat globally in obesity and diabetes with increased incidence and severity of disease (9).

The way that underlying the promotion of metabolic/endocrine disorders on the occurrence of pneumonia is not fully understood yet, but increasing evidence implies that one of the culprits is the excessive and persistent reactive oxygen species (ROS) accumulation evoked by inflammation response, which is originally executed by immune cells recruited in lungs but consequently implicated to other cells there. As known, when acting properly, inflammation response in organ and tissue is an innate response upon insults, because ROS produced by this response is the killer of various pathogens. However, if acting too intensively, inflammation response will cause excessive ROS accumulation and tissue damage in local, sometimes even cytokine storm in the whole body (10, 11), In other words, the rational ROS accumulation plays as the weapon against pathogens, but excessive ROS accumulation becomes injurer of the body itself. Therefore, keeping the intensity of inflammation and ROS accumulation at the proper level is a crucial task for pneumonia protection, including metabolic/endocrine disorder-implicated pneumonia protection. It is worthy to mention, moreover, excepting excessive ROS accumulation from acute inflammatory response, high ROS burden also exists in the organs of individuals suffering from some metabolic/endocrine disorders, such as diabetes and obesity (12–14). Generally, the chronic disease-induced ROS burden is not powerful enough to wipe out pathogens, but its consistent existent can bring about the damages in cells and tissues. Of importance, these damages provide more probabilities for pathogen invasion in the lungs, which can progress into pneumonia (15, 16).

Fortunately, innate cellular antioxidant systems also exist, acting for the blockage of over-intensive inflammation response and excessive ROS accumulation, therefore being beneficial to damage limitation and disease suppression. As the key transcription factor mediating anti-oxidant response element (ARE) associated anti-oxidative signals, nuclear factor erythroid-2-related factor 2 (Nrf2) plays an important role in eliminating ROS formed by oxidative stress (17, 18). Adenosine 5′-monophosphate (AMP)-activated protein kinase (AMPK) involves even more cellular antioxidant pathways and contributes significantly to inflammation defense (19, 20). Interestingly, recent studies report that Nrf2 and AMPK are functionally connected and play a cascade-like effect (21). Given AMPK closely links to a diverse metabolic axis in the body, it shall be reasonable to have a better understanding of the implication of oxidative stress and AMPK-Nrf2 anti-oxidative signaling in inflammatory pathogenesis, and to further explore the therapeutic potential of AMPK-Nrf2 signaling on metabolic and endocrine disorder-impacted pulmonary diseases.

We have two intentions by means of this review: to summarize the contributions of oxidative stress, either pulmonary lesions related ROS accumulation and metabolic/endocrine disorders-related ROS accumulation, to pneumonia susceptibility, and to discuss how AMPK-Nrf2 signaling contributes to maintain the metabolic and endocrine homeostasis that is the fundamental status of the body to resist pulmonary infection, to alleviate pulmonary lesions, and to prevent pneumonia.

## ROS Accumulation and Pneumonia Susceptibility

### Excessive ROS Production Caused by Pulmonary Lesions

Respiratory system exchanges with the external environment. Therefore, the lungs are easily exposed to harmful substances in the air, such as flue gas and various oxidants. Under detrimental environments, the lungs suffer mild or severe oxidative stress which results in ROS accumulation (22). ROS are the byproducts of electron transfer reactions in cells, including hydroxyl (HO^•^), superoxide anion (O^2•^−), singlet oxygen (^1^O_2_), hydrogen peroxide (H_2_O_2_), and other intermediates (23). It is worth noting that the carbonyl compounds are regarded as a new kinds of ROS, like acrolein (ACR) and methyl vinyl ketone (MVK) (24, 25).

The ROS production following pulmonary lesions results in either acute ROS increase or chronic ROS accumulation. The infection of pathogens like bacteria, viruses, and fungi, often evoke acute ROS increase. When pathogen infection occurs, immune cells infiltrate into pulmonary mesenchyme between alveolar cells and interstitial cells to respond via releasing ROS and inflammatory factors. The interaction between the pathogen and the host's immune response is intricate, and the latter sometimes acts traumatic for cells, and tissue that causes damages in the lung (26). Viewing from the molecular level, excessive ROS can react quickly and nonspecifically with the chemical groups in DNA, lipids, and proteins, resulting in cellular metabolism aberrations, severe extracellular matrix remodeling, increased mucus secretion, abnormal apoptosis and pulmonary fibrosis (10, 27, 28). Moreover, pulmonary acute ROS elevation can increase the hazard and risk of secondary lung infections, for instance, children with a history of respiratory diseases have an increased risk of reinfection pneumonia (29–31). In summary, oxidative stress is an upstream cause of pulmonary lesion, and inflammation resulted from pulmonary lesion versa aggravates oxidative stress.

As to the states of chronic ROS accumulation in the lungs, two situations are well-known. One is the sustained exposure to irritant gas that directly stimulates the upper respiratory tract, leading the injury of epithelial cells and the filtration of immune cells, then the oxidative response in the lungs including the production of large amount of ROS (32, 33). This situation is generally caused by long-term smoking, industrial pollution from the combustion of coal and petroleum, and other fuels in the atmosphere and raised dust or respirable particulate matter due to urban environmental pollution. There is also evidence that irons in the air can increase the concentration of ROS in the surface fluid of the lung epithelial cells (34). Another is chronic respiratory diseases-related ROS accumulation. Traditionally, these diseases are limited to chronic respiratory diseases, such as chronic obstructive pulmonary disease (COPD), bronchiectasis, interstitial lung disease, chronic pulmonary heart disease, and pulmonary fibrosis. As indicated by studies, a variety of immune cells in the lungs with these diseases suffer from significant oxidative stress. For example, long-term smokers and COPD patients have higher activities of alveolar macrophages, with elevated cationic protein level in eosinophil and cell surface adhesion factor and increased expression of peroxidase (MPO) in neutrophil (35, 36). Since macrophages and neutrophils are the main sources of ROS in inflammatory tissue (37), these changes occurred in cells bring about the uncontrolled ROS production and accumulation further in the lungs, together with significantly release proinflammatory mediators, an array of cytokines and metalloproteinases that participate in tissue damage (38). Moreover, it has known wildly that, disease-induced ROS elevation can continually regulate the location, modification, and complex formation of nuclear proteins, thus affecting the status of chromatin and the function of transcriptional machinery. For example, ROS can effectively promote the recruitment of RNA polymerase to the promoters of the genes encoding NF-κB- and AP)-1-responsive pro-inflammatory (39). On the other several mutations in antioxidant genes, such as GSTP1, glutathione S-transferase (GST) M1, superoxide dismutase 3 (SOD3) and microsomal epoxy hydrolase (EPHX1) is associated with the severity of COPD infection, and the function of the proteins encoded by these genes was impaired, therefore breaking the steady-state of the oxidation-reduction system and increasing the susceptibility to bacterial infections (40).

### Excessive ROS Production Caused by Endocrine/Metabolic Disorders

Despite respiratory diseases, the connection between unbalanced endocrine and metabolic circumstance and ROS accumulation starts to enter our view in the recent. ROS burden exists generally in the organs of individuals suffering from metabolic disorders, such as diabetes, obesity, and disturbances of water and electrolyte balance (14, 16). In obesity, excessive fat storage releases high level of free fatty acid (FFA) in plasma, that promotes the elevated generation of O^2•−^ from the mitochondrial electron transport chain, by inhibiting the translocation of adenine nucleotides (41). ROS can also derive from the activation of NADPH oxidase (NOX). In fact, hyperglycemia, hyperlipidemia, and angiotensin II all activate NOX, which acts to produce ROS molecules (42, 43), that remarkedly affects the status of the tissues such as adipose tissue, muscles, and liver (42, 43). What matters is that the excessive ROS can inhibit the biogenesis and dynamics of mitochondria, which in turn disrupts the homeostasis between the production and elimination of ROS. Actually, this mechanism has been revealed by studies involving both high-fat mouse models and obese patients (44, 45). For instance, in an animal study, obesity was observed to increase NOX activity but reduce the activity of antioxidant enzymes such as SOD, catalase, and glutathione in white fat tissue (46). Moreover, Importantly, as reported by Tanaka et al. (47), chronic exposure of pancreatic islets to high concentrations of glucose is toxic to beta cells, but this toxicity can be antagonized by antioxidants, N-acetyl-L-cysteine or aminoguanidine. Calcium dysregulation syndrome due to high sodium diet, low calcium diet, or insufficient vitamin D is also a type of endocrine/metabolic disorders, impaired Ca^2+^ homeostasis leads to mitochondrial dysfunction, which is characterized by changes in alterations in ATP synthesis and NADP(H) oxidation, resulting in an excessive ROS production and cell apoptosis (48). At the same time, Ca^2+^ disorders will also induce endoplasmic reticulum (ER) stress, which will cause the activation of ROS-related protein Nox4 resident on the ER (49), further lead to the accumulation of ROS.

Studies have shown that obesity and diabetes are potentially infectious with the respiratory system, such as tuberculosis, influenza and pneumococcal, staphylococcal, and opportunistic pathogens (9). Diabetes is often accompanied by the impaired innate immune system and the adaptive immune system (50, 51), one reason for Ros to clear obstacles. Moreover, leptin resistance affects the regulation of ROS in obesity which leptin increases may also regulate the accumulation of ROS in the immune response (52).

### ROS Increase Susceptibility to Pathogens

The implication of ROS to tissues largely depends on their quantities and action time, so they are a kind of double-edged sword. As mentioned in the introduction, physiologic level and action of ROS are positive for the body, contributing to antimicrobial ability. wound healing and so on (53), partially by playing roles in lymphocytes recruitment and phagocytic ability improvement (54, 55). However, if the reaction become drastic and persistent, ROS will eventually promote pathogens infections that cause tissue damage.

Excessive ROS production break immune response, either innate and adoptive. For instance, in diabetes and obesity individuals, over produced ROS influence antibody response and CD4^+^/CD8 ratio of T cells, also weaken the functions of the natural killer cells and the migration/phagocytosis of neutrophils (56–58). Moreover, oxidative stress is thought to drive impaired phagocytosis of alveolar macrophages, that closely relates to the repeated bacterial and viral infections in respiratory airways. As ROS molecules, carbonyl groups produced by various oxidative stresses, can directly cause macrophage cytoskeletal instability and, thus impair the macrophage-mediated bacterial phagocytosis (25). The interaction between carbonyl-modified extracellular matrix proteins and macrophages also inhibits the phagocytosis of macrophages (59). Consistent findings from *in vivo* study are there. For example, alveolar macrophages activation and antibacterial activity parallelly reduced in diabetic patients, accompanying the inability to deal with the challenge of M. tuberculosis (60). In COPD patients, experimental rhinovirus infection leads to elevated systemic and airway infectious inflammation (61, 62). The specific mechanism is still unclear, but some people believe that the internalization of the virus triggers the production of cellular hydrogen peroxide, and the NOX2-dependent ROS production inhibits the antiviral signaling network by modifying the Toll-like receptor 7 (63). Notably, ROS-caused damage of respiratory muscle cells has negative impact on the mechanical function of the lungs, which is important for breathing and gas exchange, therefore being another force to disturb the health of the lungs and to increase the susceptibility of this organ to pathogen infection (64).

On the other hand, ROS accumulation creates an environment suitable for pathogen settlement. One of the bacterial survival strategies depends on the ability to form biofilms and establish a special community. It has been reported recently that ROS accumulation can increase the variability of biofilm formation and induce the antibiotic resistance of *Pseudomonas aeruginosa* (65). Clinical evidence from patients with diabetes and cardiovascular diseases, collected more recently, have confirmed this notion (66). It is suggested that, when biofilm changed, the host is difficult to neutralize bacterial pathogens, which leads the colonization of bacteria becomes easier in the airway epithelial cells, herein resulting in infectious pneumonia, even severe acute respiratory distress syndrome (ARDs) and sepsis (67).

One more point that needs to be mention is that the accumulation of ROS can accelerate cellular aging, so-called senescence, at least partially through the prompted secretion of proinflammatory cytokines and proteases, companied with the oxidative response and produced by inflammatory cells. Importantly, studies have shown that Keratin 10, laminin receptor, and PAFR are highly expressed in senescent cells, and these proteins are ligands for pneumonia-associated bacteria (68). Oxidative stress-induced senescence is connecting with the raise of inflammation response. This notion comes from the study demonstrating that Oxidative stress and senescence are significantly responsible for the over-activated expression of pro-inflammatory cytokines (69). Interestingly, with high ROS-induced intracellular DNA damage, the innate anti-viral pathway, cGAS-STING, in these cells did not elevate the expression of the IFN1 family genes, while that will be observed in normal cells. It suggests a possibility that the endogenous ROS accumulation probably can weaken the power of the innate immune pathway, at least in certain case, toward DNA virus infection. The recent emergence of SARS-CoV-2 related coronavirus COVID-19 and influenza B virus has made respiratory viruses an important pathogen for infectious pneumonia (70, 71). A recent meta-analysis has shown that the most common comorbidities of COVID-19 clinical symptoms are hypertension and diabetes, followed by cardiovascular diseases and respiratory diseases (72). Statistics also show that patients with diabetes and influenza have more COVID-19 infections (73, 74). This result reveals that the high ROS environment of the body caused by various diseases increases the risk of viral infection. This persistent pandemic threat makes the identification and development of new treatment strategies, especially the treatment of infectious pneumonia, an urgent matter.

### ROS Clearance Alleviates Pathogen Susceptibility

The body relies on an antioxidant system to remove ROS and maintain redox homeostasis. According to their molecular structure and mechanism of action, antioxidants in organisms can be divided into: (1) antioxidants involved in ROS captures, such as glutathione (GSH), vitamin C and E; (2) Enzyme active substances participated in the antioxidant pathway, including superoxide dismutase (SOD), catalase, glutathione oxidase (GPx), and glutathione reductase (GR) (75). For example, GSH can be combined with hydrogen peroxide (H_2_O_2_) and is oxidized to oxidized glutathione (GSSG), in turn, GSSG can be reduced to GSH by glutathione reductase and re-participate in body metabolism. In addition, H_2_O_2_ also can be converted to water by mitochondrial glutathione peroxidase (GPx) (76, 77). Clinical studies have shown that taking high doses of vitamin C can alleviate endothelial tissue damage caused by ROS production from ischemia and reduce the risk of bacterial infection in patients after ischemia/reperfusion (78). Moreover, vitamin C supplementation may prevent community-acquired pneumonia, but the prophylactic use of vitamin C to prevent pneumonia in the general population deserves more researches and evidences (79). Another research in the acute exacerbation of chronic obstructive pulmonary disease (AECOPD), use of GPx mimic Ebselen and Apocynin (a NOX2 oxidase inhibitor) can effectively reduce the excess ROS produced and reduces inflammation caused by influenza A (80).

Recent studies have shown that intervention of ROS clearance by external drugs can effectively prevent pathogen infection. For instance, N-acetyl-L-cysteine (NAC) is a commonly antioxidant which has been used in some antiviral models. NAC can inhibit the replication of related viruses, such as HIV (81, 82), Influenza A (83, 84), RSV (85), PCV2 (86), alleviate susceptibility of viruses. Resveratrol as an antioxidant also has antibacterial activity against *Staphylococcus aureus, Bacillus cereus*, and others (87–89). A 2012 study showed that combined use of resveratrol and antibiotics has a greater antibacterial effect than single-use (90). But, researchers reported that resveratrol combined with levofloxacin inhibits the antibacterial activity, levofloxacin perform bactericidal effect through ROS activation while resveratrol inhibits it (91). The mechanism of resveratrol eliminates ROS for antibacterial is not clear, but resveratrol's own strong antioxidant properties and its ability to inhibit the diffusion of biofilms (92, 93) have given the possibility of an investigation. Besides, it has also been reported that antioxidants have the same molecular structure as some antibacterial drugs, such as vanillin, resveratrol, curcumin, may be a possibility for a new antibacterial agent to address antibiotic resistance (94).

For a long time, people have generally believed that ROS is a weapon for the immune system against pathogens. However, the use of antioxidants also is one of the treatments for infections. We can speculate that the long-accumulated ROS and the ROS of the immune response are different. This difference may exist in production, regulatory mechanisms, chemical composition, and others, which needs further exploration.

## The Role of AMPK-Nrf2 Pathway in Pneumonia

### AMPK—an Upstream Regulator of Antioxidative Response

AMPK is a serine/threonine kinase in all eukaryotic tissues and organs, can be activated by various stimuli that affected cell metabolism, including hypoxemia, alimentary deficiency, exercise, and many hormones and substances. The ratio of AMP to ATP mainly regulated AMPK, The activation of AMPK requires the binding of AMP and ADP to the γ subunit and the β subunit myristoylation to promote phosphorylation at Thr172 (95). If the AMP content is excessive (3–5 folds), the myristoylation β subunit is not required to directly activate AMPK (96, 97). In addition, Ca^2+^/calmodulin-dependent protein kinaseβ (CaMKKβ), kinase serine/threonine kinase 11 (LKB1), and transforming growth factor-β-activated kinase 1 also can phosphorylation of Thr172 to activate AMPK (95). In addition, the activation of AMPK also closes the anabolic pathway, like a catabolic switch (98). The biological functions of AMPK mainly include: stimulate fatty acid oxidation of liver and skeletal muscle, promote muscle glucose uptake, inhibit the production of triglyceride, cholesterol, adipose, inhibit lipolysis of adipocytes, and regulate pancreatic insulin secretion (95, 99, 100). Overall, AMPK is a key balancer of energy supply and demand and a key integrator of important pathways such as redox, inflammatory production, and autophagy in cells.

Most previous studies have shown that metabolic disorders can seriously affect AMPK activity. For example, in diabetic patients, the AMPK activity of glomerular epithelial cells in a high glucose environment is significantly reduced, and the glomerular epithelial cells are significantly proliferated and hypertrophic (101). Similarly, in pancreatic β cells, the high glucose environment significantly reduced AMPK activity. However, it is worth noting that activating AMPK activity in pancreatic β-cells can inhibit glucose-stimulated insulin release, and that AMPK activation can promote the increase of ROS levels in β-cells, while inhibiting the expression of glucokinase, and ultimately induce β-cell apoptosis (102). In type 2 diabetes, the AMPK to its downstream glucose transporter-4 (GLUT4) signaling pathway is impaired in patients' skeletal muscles. The LKB1-AMPK-GLUT4 signaling pathway is an important signaling pathway that promotes skeletal muscle glucose uptake, transport, and utilization (103, 104). In skeletal muscle of obese individuals, the uptake rate of long-chain fatty acids (LCFA) was significantly higher than normal (105), and the plasma membrane protein FAT/CD36, which plays a key role in LCFA transport, was found to be highly expressed in the myotubes of obese patients (106). More importantly, activating AMPK with the drug AICAR can induce all LCFAs to transfer to the plasma membrane (107). Studies of other signaling cascade pathways have shown that inhibition of extracellular signal receptor kinase (ERK1/2) can prevent FAT/CD36 translocation (108, 109), and stimulation of protein kinase C can induce the shift of FAT/CD36. The internal Ca^2+^ concentration is related to signal transport, and subsequent further research proves that CaMKK located upstream of AMPK mainly affects LCFA transport (110). Because glucagon activates AMPK and insulin inhibits AMPK, this indicates that AMPK participates in the endocrine feedback loop and can respond to feedback regulation of hormone (such as insulin) release (111), which is necessary to respond to nutrient and energy stress. But hormones have opposite effects on AMPK activity in the central and peripheral circuits (112). Therefore, we can reasonably speculate that excessively transported LCFA can also inhibit AMPK activity.

Several studies have shown that activation of AMPK can inhibit oxidative stress triggered by different lesions, hyperglycemia, and hyperlipidemia. For example, in hyperglycemic conditions, AMPK activation induces the expression of mitochondrial uncoupling protein 2 (UCP-2), which significantly reduces the production of superoxide free radicals in mitochondrial of hyperglycemic endothelial cells (113). On the other hand, there is emerging evidence that AMPK can inhibit the activation of the NF-κB system (a key class of immune stress-related nuclear transcription factors) through its signaling network, thereby blocking the transcription of innate immunity and inflammation-related factors (114).

Previous studies have shown that the AMPK-FOXO3-Thioredoxin (Trx) is an antioxidant pathway (115). Trx is a disulfide reductase which protects cellular proteins from cysteine oxidation. Under physiological conditions, Trx binds to thioredoxin interacting protein (Txnip) and functions are inhibited (116). When it comes to oxidative stress, the complex between Trx and Txnip dissociates, then Txnip bind to the inflammatory hormone receptor Nod-like receptor protein 3 (NLRP3) receptor, triggering the expression of a variety of downstream inflammatory factors (117). Meanwhile, Trx regulated by AMPK can play an antioxidant role under oxidative stress situation. Experiments have shown that the injection of AICAR in ApoE^−/−^ mice induced by high-fat increased Trx expression of the aortic wall, accompanied with ROS decreased (116) Moreover, AMPK downstream protein mTOR, a nutrient and growth factor sensing complex, by phosphorylating eukaryotic translation initiation factor 4E (eIF4E) and ribosomal protein S6 kinase (S6K) to control protein synthesis (118, 119). Protein anabolism is positively correlated with ROS production (120). The activation of AMPK can inhibit the activity of mTOR1, reducing anabolism-related ROS accumulation.

Furthermore, the PPAR proteins family, as ligand-dependent transcriptional regulators, are closely linked to antioxidants. Researches have shown that PPARδ is downstream of AMPK through enhance antioxidant capacity to protect endothelial function from ER stress and oxidative stress (121), such as increase Sirt1 activity and NO production (121, 122). Previous studies have shown that PPARγ in elderly dermal fibroblasts (HDF) reduces the expression of pro-inflammatory cytokines (123). Moreover, Ligand-activated PPARδ also blocked angiotensin II (Ang II), inhibiting ROS production (124).

### Nrf2—a Key Antioxidant Transcription Factor

The activation of the Nrf2-antioxidant response element (ARE) signaling pathway is the main antioxidant means of the cell. This pathway regulates protein produce to eliminate active oxidants by group binding or enzyme reaction. A variety of chronic diseases, including neurodegenerative diseases, metabolic diseases, and cardiovascular diseases, are closely related to Nrf2 and serve as potential therapeutic targets (125). In normal conditions without stress, Nrf2 is retained in the cytoplasm, mainly because Nrf2 binds to the E3 ubiquitin ligase substrate adaptor [Kelch-like ECH-related protein 1 (Keap1)], which makes Nrf2 fast degrade by ubiquitination.

Keap1 is highly sensitive to oxidants and electrophiles. Under the oxidative stress, The cysteine residue of Keap1 combined with oxidants and be modified, thereby releasing Nrf2 that binds to Keap1, resulting in the accumulation of Nrf2 in the nucleus and activation of transcription of the ARE-related genes, which coding the major detoxication enzymes, including Heme Oxygenase-1 (HO-1), NADPH quinone oxidoreductase 1 (NQO1), and GSH redox system-related enzymes, such as γ-glutamyl cysteine synthetase (γ-GCS) subunit GCLC, glutathione peroxidase (GPx), glutathione reductase (GSR), and glutathione-S-transferase (GSTs), prevent oxidative damage by increasing the synthesis of reduced GSH, free radicals are scavenged and the cell redox balance is maintained (126–129).

The loss or inactivation of Nrf2 can induce a variety of inflammatory-related diseases. In experimental diabetic mouse models, Nrf2 deficiency increases oxidative stress and induces ROS accumulation, leading to early heart damage and cardiovascular dysfunction (mainly increased cardiac fibrosis). Treatment with Nrf2 inducers (such as sulforaphane and myricetin) can effectively protect the mouse heart from damage caused by high ROS (130). In humans, the transcriptional activity of Nrf2 affects the occurrence of pulmonary diseases. For example, the transcriptional characteristics of Nrf2 in alveolar macrophages of smoking-induced emphysema patients are lower than normal (131). Moreover, Nrf2^−/−^ mice exhibit enhanced susceptibility to cigarette smoke-induced emphysema (132).

### AMPK Mediates Antioxidative Cascade by Activating Nrf2

Recent studies have shown that AMPK can directly phosphorylate with Nrf2. In cells, there is phenomenon display that activation of AMPK leads to migration and aggregation of transcription factor Nrf2 into the nucleus. AMPK phosphorylates Nrf2 located in the Ser558 residue (Ser550 in mice) in the normal nuclear export signal, a result confirmed from an *in vitro* kinase and peptide competition assays (21), and the nuclear import effect of Nrf2 is lost in the case of the Ser550A mutation, even under AMPK activation. Moreover, it has been reported that AMPK inhibits the activity of glycogen synthase kinase-3 (GSK3β) (133). Specific activation of GSK3β can significantly reduce nuclear Nrf2 levels. GSK3β inhibits Nrf2 activity through phosphorylation and nuclear exclusion (134). Therefore, it can be considered that AMPK indirectly reduces the level of Nrf2 in the nucleus by inhibiting the activity of GSK3β.

Numerous pharmacological experiments have also shown that activating the AMPK-Nrf2 pathway can effectively alleviate many inflammatory-mediated diseases. Compounds HP-1c and NBP, which are used in brain inflammation, can prevents cerebral ischemia, reduces neurotoxicity, and active angiotensin II receptor blockers (ARBs) to establish neuroprotective effects on neurodegenerative diseases (135) by activating AMPK. In addition, AMPK activating also promotes the conversion of microglia from pro-inflammatory M1 to anti-inflammatory M2, thereby reducing the inflammatory response in brain (136). It is known that ARBs and NBP can restore and activate Nrf2 expression, either cause AMPK activation to alleviate inflammation (136), therefore it can be speculated that there may be a crosstalk between AMPK and Nrf2. About other activation pathways of AMPK to Nrf2, previous studies have shown that the cysteine residues (C151) in Keap1 were essential for inhibiting nuclear translocation of Nrf2 (137). Moreover, some studies suggested that some compounds, such as TBHQ, sulforaphane, and quercetin, could modify the cysteine residue in Keap1 (138), also activate AMPK-related pathways.

### AMPK-Nrf2 Activation Ameliorates Pneumonia

There are many pieces of evidences confirm that activation of the AMPK-Nrf2 pathway can alleviate pneumonia-related symptoms. As an agonist of AMPK, BBR down-regulates the expression of pro-inflammatory genes in the lungs of mice injected with LPS and up-regulates the expression of Nrf2 targeting genes. Besides, this effect of BBR is impaired in Nrf2 gene-deficient mice. Furthermore, in an Nrf2-depleted mice model, BBR prolonged the survival time of endotoxin-stimulated mice, and its effect on plasma redox regulation was greatly attenuated (139).

Enhancing the activity of Nrf2 can play the same role. Isothiocyanate thiocyanate (SFN) activation of Nrf2 restores bacterial recognition and phagocytosis, enhances the ability of alveolar macrophages to clear lung bacteria, and reduces lung inflammation in wild-type mice exposed to cigarette smoke, while mice with knockout Nrf2 are not (140). Syndecan 4, a transmembrane proteoglycan, could increase the number of bronchiolar progenitor cells that have subsided due to inflammation by dedifferentiation of pulmonary club cells into bronchiolar progenitor cells, and its mechanism is mainly through regulating Nrf2 activity (141, 142). In recent years, it has been proved that the normal function of Nrf2 is one of the key factors to ensure the homeostasis of airway epithelial health. Keap1-Nrf2 system is helpful for the lineage-specific differentiation of stem and progenitor cells, which play an important role in the stable operation of various types of cells in the lung (143).

Activation of the AMPK-Nrf2 pathway can also prevent or relieve pneumonia indirectly by alleviate the symptoms of obesity and diabetes. Surveys have shown that the mortality and morbidity of pneumococcal pneumonia and influenza in obese and diabetic patients are generally higher (9), and that the mortality of pneumonia-related surgery in diabetic patients is higher (144). Therefore, it is reasonable to believe that obesity or diabetes caused by metabolic disorders inhibits AMPK-Nrf2 pathway activity, just as free fatty acid accumulation and high glucose environment are accompanied with oxidative stress damage and ROS accumulation (145). The use of drugs to activate AMPK, including AICAR (146), metformin (147), TZDs (148), berberine (149), and resveratrol (150), can activate skeletal muscle AMPK, improve insulin sensitivity, improve T2D symptoms, and promote lipolysis. It has also been reported that in T2D model mice treated with cassia seed extract, the mRNA and protein expression levels of P-LKB1, P-AMPKa12, AMPKa2, P-AMPKa2, GLUT4 are increased, which means the glucose transport pathway is restored, results in glutathione levels increased and ROS levels decreased significantly (151). However, the detailed molecular mechanism of mediation of insulin sensitization directly through AMPK-Nrf2-ROS is unclear. To summarize, the activation of AMPK-Nrf2 reduces the accumulation of ROS in the lungs, especially in the case of severe oxidative stress in body tissues such as obese or diabetic patients. We can use this new mechanism to discover new treatment strategies to prevent and improve the occurrence of pneumonia.

## Conclusion and Perspectives

**Figure 1:**
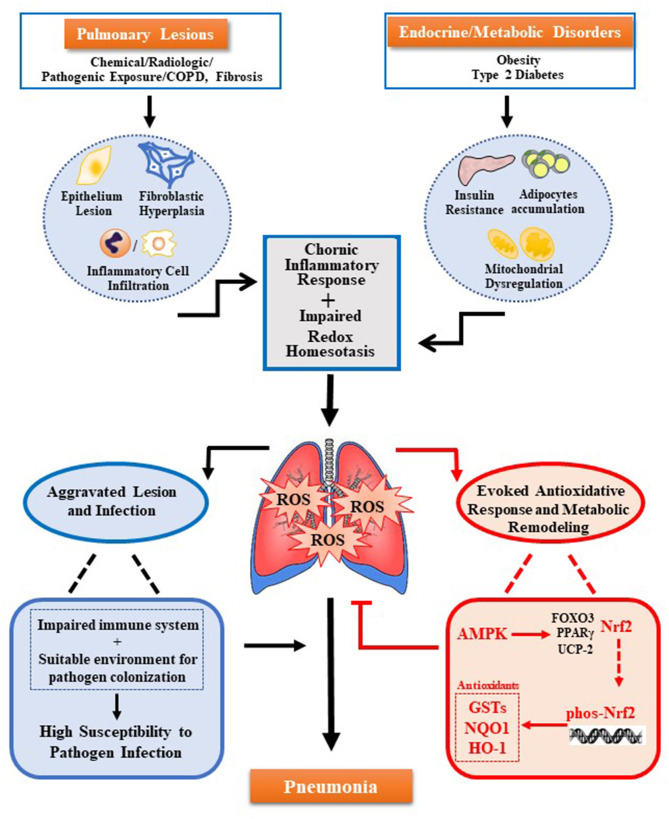
Multiple oxidative stress factors induce pneumonia and AMPK-Nrf2 pathway inhibits pulmonary ROS accumulation. Pulmonary ROS accumulation mainly caused by pulmonary lesions and endocrine/metabolic disorders. Trigger factors include chronic inflammation response induced by damage of pulmonary cells (alveolar epithelial cells, alveolar cells, lung fibroblasts, and others) and infiltration of immune cells in the lungs, as well as the impaired redox homeostasis induced by metabolic disease-related insulin resistance, adipocytes accumulation, and mitochondrial dysfunction. Accumulated ROS can cause damage to the immune system and colonization of pathogens, both of which increase pulmonary susceptibility and cause pneumonia. Meanwhile, ROS can also trigger several antioxidant pathways regulated by AMPK. Among them, the antioxidant effect of AMPK-Nrf2-AREs plays a key role. The activation of this pathway can inhibit the accumulation of ROS in the lungs to prevent or alleviate pneumonia.

Pulmonary lesions and metabolic disorders can cause oxidative stress and accumulation of ROS, which further remodeling the pulmonary microenvironment. These damages result in the impaired immune system and increases colonization of pathogens, to raise the susceptibility of pneumonia. The new molecular mechanism reveals that AMPK-Nrf2 has an antioxidant cascade effect, and the regulation of the AMPK-Nrf2 pathway to eliminate pulmonary ROS can prevent or improve pneumonia. As shown in the figure (see Figure 1), the use of AMPK activators has been increased for the treatment of various inflammations. However, for many chronic inflammations, especially in the lungs constantly exposed to high levels of oxidative stress, the body's antioxidant activity is crucial. Current research has revealed that Nrf2 is located downstream of AMPK and has a direct effect on the inflammatory response. In addition, promoting the phosphorylation of Nrf2 through AMPK by pharmacological or genetic methods to alleviate the accumulation of excess ROS in the body may be a potential therapeutic strategy. Therefore, the regulation of the activity of the AMPK-Nrf2 pathway has great research prospects for the treatment of pneumonia.

## Author Contributions

WX and TZ mainly performed the review writing and revision. HX guided the writing planning and manuscript preparation. All authors contributed to the article and approved the submitted version.

## Conflict of Interest

The authors declare that the research was conducted in the absence of any commercial or financial relationships that could be construed as a potential conflict of interest.
